# Clinical and optical coherence tomography predictors of spontaneous resolution of diabetic macular edema: incremental value of the subfoveal large choroidal vessel layer thickness ratio

**DOI:** 10.3389/fmed.2026.1901761

**Published:** 2026-07-30

**Authors:** Xiangqiang Lin, Yun Xie, Yingying Chen

**Affiliations:** 1 Department of Ophthalmology, Qingdao Chengyang People’s Hospital, Qingdao, Shandong, China; 2 Department of Pediatrics, Huai’an Clinical Medical College of Jiangsu University (Huai’an Hospital of Huai’an City), Huai’an, Jiangsu, China; 3 Department of Endocrinology and Metabolism, Xinchang County People’s Hospital, Shaoxing, Zhejiang, China

**Keywords:** choroidal biomarker, diabetic macular edema, optical coherence tomography, prediction model, spontaneous resolution

## Abstract

**Background:**

Spontaneous anatomical improvement can occur in selected patients with diabetic macular edema (DME) managed initially by observation, but reliable predictors remain insufficiently defined. This study evaluated clinical and optical coherence tomography (OCT) predictors of 6-month spontaneous resolution and the incremental prognostic value of the subfoveal large choroidal vessel layer thickness ratio (SLCVLTR).

**Methods:**

This retrospective single-center study included 302 eyes of 302 adults with type 2 diabetes and OCT-confirmed DME who underwent initial observation without immediate DME-directed treatment from March 2021 to March 2025. Spontaneous resolution was defined as both a ≥20% reduction in central subfield thickness (CST) and an absolute CST ≤ 300 μm at 6 months without DME-directed treatment. Multivariable logistic regression identified independent predictors. Discrimination, calibration, and clinical utility were assessed using receiver operating characteristic analysis, the Hosmer–Lemeshow test, and decision curve analysis.

**Results:**

Spontaneous resolution occurred in 83 eyes (27.5%). Higher hemoglobin A1c (odds ratio [OR], 0.762; 95% confidence interval [CI], 0.597–0.971), greater CST (OR, 0.989; 95% CI, 0.983–0.995), cystoid versus diffuse edema (OR, 0.438; 95% CI, 0.232–0.826), more hyperreflective foci (OR, 0.921; 95% CI, 0.872–0.973), and higher SLCVLTR (OR, 0.932; 95% CI, 0.895–0.972) were independently associated with lower odds of spontaneous resolution, whereas intact ellipsoid-zone continuity was associated with higher odds (OR, 1.855; 95% CI, 1.028–3.347). Adding SLCVLTR to the clinical–retinal OCT base model increased the area under the curve from 0.812 to 0.847 (*p* = 0.027). The combined model showed good calibration (Hosmer–Lemeshow *p* = 0.935) and the highest net benefit across threshold probabilities of 10–60%.

**Conclusion:**

Spontaneous resolution of DME is associated with systemic metabolic control, edema severity, retinal microstructural integrity, and choroidal laminar structure. SLCVLTR provides independent and incremental prognostic information and may improve risk stratification in carefully selected patients considered for initial observation.

## 1 Introduction

Diabetic macular edema (DME) is a major cause of visual impairment among working-age individuals with diabetic retinopathy, and its prevalence increases with diabetes duration, retinopathy severity, and systemic metabolic burden (1). Current understanding of DME emphasizes chronic hyperglycemia-related disruption of the inner blood-retinal barrier, leakage from the superficial and deep retinal capillary plexuses, vascular endothelial growth factor (VEGF)-mediated increases in vascular permeability, inflammation, oxidative stress, and neurovascular unit dysfunction (2). These processes collectively lead to intraretinal and/or subretinal fluid accumulation and diverse optical coherence tomography (OCT) morphological patterns (3). Intravitreal anti-VEGF therapy is a principal treatment for center-involved DME, but heterogeneous treatment response, the long-term burden of repeated injections, and real-world adherence to follow-up continue to affect individualized management (4). In clinical practice, some patients with relatively stable visual acuity and the capacity for close monitoring may remain stable or show anatomical improvement during initial observation, suggesting the need to identify patients likely to undergo short-term spontaneous resolution (5).

The natural course of DME is jointly influenced by systemic metabolic status, retinal edema burden, and retinal microstructural integrity. Higher hemoglobin A1c (HbA1c), markers of renal microvascular injury, and longer diabetes duration have been shown to be associated with the development and progression of DME (6). OCT biomarkers, including central subfield thickness (CST), macular edema subtype, outer retinal integrity, hyperreflective foci (HRF) burden, and disorganization of the retinal inner layers (DRIL), are also closely related to anatomical or visual outcomes (7). However, reliance on a single retinal thickness metric alone does not fully explain interpatient differences in edema absorption capacity.

The choroid supplies the retinal pigment epithelium (RPE) and outer retina, and alterations in choroidal structure may modulate the course of DME through the outer retinal/choroidal microenvironment. Recent studies using enhanced-depth imaging optical coherence tomography (EDI-OCT), swept-source/spectral-domain OCT, and optical coherence tomography angiography (OCTA) have shown that subfoveal choroidal thickness, the choroidal vascularity index (CVI), laminar choroidal structures such as Haller’s/Sattler’s layers, and the associations between OCTA-derived microvascular metrics and choroidal metrics may be altered in diabetic eye disease (8–11). In addition, sex- and age-related differences in choroidal thickness in healthy individuals suggest that relative laminar indices may more effectively reduce the influence of interindividual variability than single absolute thickness measurements (12). The subfoveal large choroidal vessel layer thickness ratio (SLCVLTR) reflects the relative proportion of Haller’s layer (large choroidal vessel layer) within the total subfoveal choroidal thickness; this relative laminar index can characterize the proportion of the large choroidal vessel layer and may capture imaging information related to fluid transport around the outer retina and retinal pigment epithelium (RPE)-Bruch’s membrane complex (10, 11). This study comprehensively evaluated the associations of clinical factors, retinal OCT parameters, and SLCVLTR with spontaneous resolution of DME and analyzed the incremental predictive value of SLCVLTR beyond existing clinical and retinal OCT models.

## 2 Materials and methods

### 2.1 Study population

This retrospective cohort study collected clinical data from patients diagnosed with DME in the Department of Ophthalmology, Qingdao Chengyang People’s Hospital, from March 2021 to March 2025. The study was approved by the Ethics Committee of Qingdao Chengyang People’s Hospital (approval No. 2026042902). Because this was a retrospective observational study using only deidentified clinical data, the ethics committee waived the requirement for written informed consent. The study adhered to the principles of the Declaration of Helsinki.

This study included only patients with type 2 diabetes mellitus (T2DM). Type 1 diabetes mellitus (T1DM) and T2DM may differ in age at onset, disease-duration distribution, degree of insulin dependence, systemic metabolic background, rate of retinopathy progression, and choroidal structural parameters; restricting the cohort to T2DM helps reduce clinical heterogeneity. T2DM was diagnosed according to internationally accepted diagnostic criteria for diabetes (13). Diabetic retinopathy was staged according to the International Clinical Diabetic Retinopathy Disease Severity Scale (14).

The inclusion criteria were as follows: (1) diagnosis of T2DM; (2) OCT-confirmed DME with CST > 300 μm; (3) no immediate intravitreal anti-VEGF injection, intravitreal corticosteroid injection, or macular laser photocoagulation after the first diagnosis of DME, with at least 6 months of observational follow-up; (4) good-quality baseline and follow-up OCT images (signal strength index ≥6), with clearly discernible choroidal laminar structures; and (5) age ≥18 years.

The exclusion criteria were as follows: (1) other ocular diseases that could cause macular edema, such as retinal vein occlusion, uveitis, or Irvine-Gass syndrome; (2) history of vitreoretinal surgery; (3) intravitreal anti-VEGF or corticosteroid injection within the previous 6 months; (4) high myopia, defined as spherical equivalent refractive error ≤−6.00 D or axial length ≥26.5 mm; (5) significant media opacity affecting OCT image quality; (6) inability to clearly identify the choroid-scleral interface on OCT; and (7) incomplete clinical data. After applying the inclusion and exclusion criteria, 302 patients (302 eyes) were included. If both eyes met the eligibility criteria, the eye with the higher baseline CST was included; if baseline CST was equal in both eyes, the eye with worse best-corrected visual acuity (BCVA) was included.

### 2.2 Definition of spontaneous resolution and treatment handling

Initial observation in this study was based on real-world clinical decision-making. Included patients did not receive immediate anti-VEGF therapy, intravitreal corticosteroid therapy, or macular laser therapy after the initial diagnosis of DME and had 6-month endpoint assessment data. Initial observation was generally based on relatively stable visual acuity, absence of tractional maculopathy on OCT, absence of fovea-threatening structural changes requiring urgent intervention, ability to attend follow-up every 4–8 weeks, the need for simultaneous optimization of systemic metabolic status, and patient preference to defer injection therapy after adequate communication (15). DME management strategy and rescue treatment selection followed clinical guidelines, physician judgment, and patient preference (16).

Spontaneous resolution of DME was defined as a decrease in CST of at least 20% from baseline at the 6-month endpoint assessment without DME-directed treatment and recovery to an absolute CST ≤ 300 μm; both criteria had to be met. If a patient received rescue treatment for macular edema due to DME progression before the 6-month endpoint assessment, including anti-VEGF therapy, intravitreal corticosteroid therapy, or macular laser, the eye was assigned to the non-resolution group, and the most recent pretreatment OCT record was used as evidence of disease progression. Patients who underwent peripheral panretinal photocoagulation (PRP) for proliferative diabetic retinopathy (PDR) or high-risk PDR during follow-up but did not receive DME-directed treatment were retained in the primary analysis; peripheral PRP was considered a non-DME-directed treatment, and robustness was evaluated by sensitivity analysis excluding PRP cases during follow-up.

### 2.3 Clinical data collection

Baseline clinical data were collected for all patients, including: (1) demographic characteristics: age, sex, and body mass index (BMI); (2) diabetes-related information: diabetes duration, HbA1c, fasting plasma glucose, and insulin use; (3) systemic comorbidities: history of hypertension and history of dyslipidemia; (4) laboratory results: serum creatinine, blood urea nitrogen, lipid profile (total cholesterol, triglycerides, low-density lipoprotein cholesterol [LDL-C], and high-density lipoprotein cholesterol [HDL-C]), and urinary albumin-to-creatinine ratio (UACR); and (5) ophthalmic data: BCVA recorded as the logarithm of the minimum angle of resolution (LogMAR), intraocular pressure, lens status, diabetic retinopathy (DR) stage, and history of PRP.

### 2.4 OCT examination and image analysis

All patients underwent macular scanning using spectral-domain optical coherence tomography (SD-OCT; ZEISS CIRRUS HD-OCT, Carl Zeiss Meditec, Dublin, CA, USA). Horizontal and vertical enhanced-depth imaging (EDI) mode scans centered on the fovea were acquired. The scan length was 9 mm, with a scan spacing of 30 μm; 20 B-scans were acquired in each direction, and at least 25 frames were averaged for each scan line to obtain high-quality images. Manual measurements were performed using the built-in measurement software of the ZEISS CIRRUS HD-OCT system.

**Figure 1:**
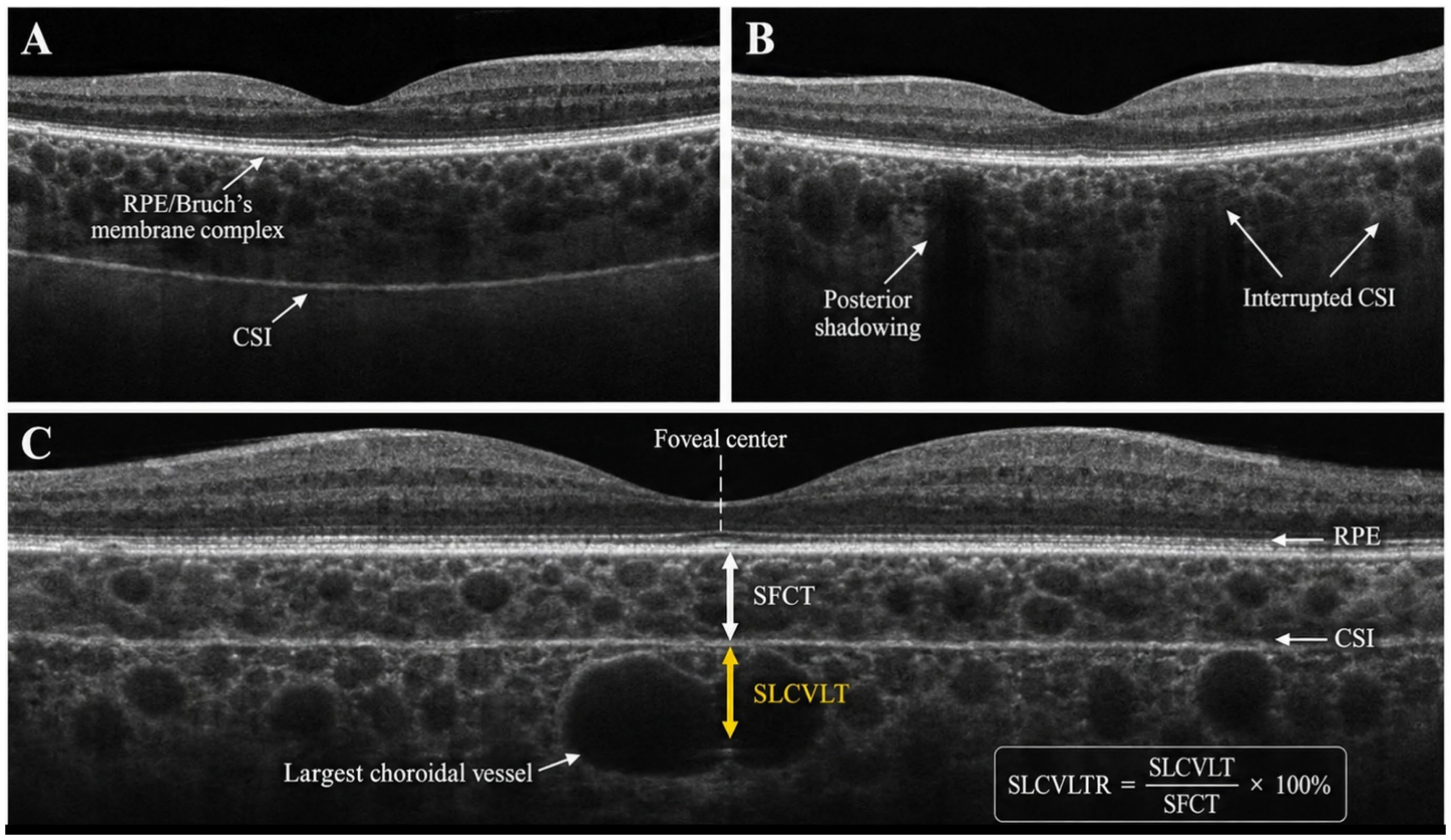
Measurement of the choroid-scleral interface (CSI) and subfoveal large choroidal vessel layer thickness ratio (SLCVLTR) on enhanced-depth imaging optical coherence tomography (EDI-OCT). **(A)** Representative image with a clearly discernible CSI, in which both the retinal pigment epithelium (RPE)/Bruch’s membrane complex and CSI are continuously visible; **(B)** Example judged to have an indistinct CSI because of posterior shadowing and boundary interruption; **(C)** Subfoveal choroidal thickness (SFCT) is the vertical distance from the outer border of the RPE to the CSI, and subfoveal large choroidal vessel layer thickness (SLCVLT) is the vertical distance from the CSI to the boundary of the largest subfoveal choroidal vessel toward Sattler’s layer. SLCVLTR = SLCVLT/SFCT × 100%. EDI-OCT, enhanced-depth imaging optical coherence tomography; CSI, choroid-scleral interface; RPE, retinal pigment epithelium; SFCT, subfoveal choroidal thickness; SLCVLT, subfoveal large choroidal vessel layer thickness; SLCVLTR, subfoveal large choroidal vessel layer thickness ratio. RPE/Bruch’s membrane complex = RPE/Bruch’s membrane complex; Posterior shadowing = posterior shadowing; Interrupted CSI = interrupted CSI; Foveal center = foveal center; Largest choroidal vessel = largest choroidal vessel.

A clearly discernible choroid-scleral interface (CSI) was defined as follows: on a horizontal EDI-OCT B-scan passing through the foveal center, both the RPE/Bruch’s membrane complex and CSI were continuously identifiable; the CSI appeared as a relatively continuous hyperreflective outer choroidal boundary over at least 1.5 mm centered on the fovea; the vertical measurement line through the fovea showed no obvious vascular shadowing, motion artifact, or media-opacity-related boundary interruption; and both graders were able to complete measurements of subfoveal choroidal thickness and subfoveal large choroidal vessel layer thickness (Figure 1).

Subfoveal choroidal thickness (SFCT) was defined as the vertical distance from the outer border of the RPE to the CSI at the foveal center. Subfoveal large choroidal vessel layer thickness (SLCVLT) was defined as the vertical distance below the foveal center from the CSI to the boundary of the largest choroidal vessel toward Sattler’s layer. SLCVLTR was calculated as SLCVLT/SFCT × 100% and represents the relative proportion of the large choroidal vessel layer within the total SFCT.

CST represented the mean retinal thickness within the central 1-mm circle of the Early Treatment Diabetic Retinopathy Study (ETDRS) grid. Macular edema subtype was classified as diffuse retinal thickening (DRT), cystoid macular edema (CME), or serous retinal detachment (SRD). Outer retinal integrity included ellipsoid zone (EZ) continuity and external limiting membrane (ELM) integrity. EZ continuity was assessed within the central 3-mm region of the horizontal B-scan passing through the fovea; a continuously discernible EZ hyperreflective band within this region was classified as intact EZ, whereas focal interruption or loss of continuity was classified as EZ disruption.

HRF were defined as well-circumscribed punctate or small round hyperreflective signals located within the neurosensory retina, with reflectivity similar to or greater than that of the retinal nerve fiber layer, a maximum diameter ≤30 μm, and no obvious posterior shadowing. Lesions >30 μm, patchy or confluent hyperreflective lesions, lesions predominantly located in the outer plexiform layer/outer nuclear layer, lesions with obvious posterior shadowing, or lesions corresponding to clinical hard exudates on color fundus photography were not counted as HRF (17). HRF counts were performed within the central 3-mm region of the horizontal B-scan passing through the fovea (18). HRF counts recorded lesions within the neurosensory retina that met the above criteria; choroidal-layer reflectivity signals were used for SFCT and SLCVLT structural measurements. DRIL was defined as the inability to continuously identify any boundary among the ganglion cell-inner plexiform layer complex, inner nuclear layer, and outer plexiform layer within the central 1-mm assessment window of the horizontal B-scan passing through the fovea, with a continuous horizontal extent of unidentifiable boundaries ≥500 μm. The 500-μm threshold corresponds to involvement of 50% of the central 1-mm assessment window and was based on the central 1-mm measurement framework used in previous DME-related DRIL studies assessing the association between foveal DRIL and visual function, while also taking into account the reproducibility of binary grading (19–21). DRIL as an OCT biomarker related to visual function in DME has also been supported by subsequent imaging and mechanistic studies (22).

### 2.5 Statistical analysis

Statistical analyses were performed using SPSS 26.0 (IBM Corp., Armonk, NY, USA) and R 4.3.0 (R Foundation for Statistical Computing, Vienna, Austria). Normally distributed continuous variables are presented as mean ± standard deviation, and between-group comparisons were performed using independent-samples *t* tests. Non-normally distributed continuous variables are presented as median (first quartile, third quartile) and were compared using the Mann–Whitney *U* test. Categorical variables are presented as counts (percentages) and were compared using the *χ*^2^ test or Fisher exact test. A two-sided *p* < 0.05 was considered statistically significant.

Spontaneous resolution of DME was used as the dependent variable. Variables with *p* < 0.10 in univariate analyses and variables considered clinically important were included in multivariable logistic regression to identify factors independently associated with spontaneous resolution of DME. To avoid including excessive irrelevant variables while preserving clinical relevance, candidate variables were pre-screened; least absolute shrinkage and selection operator (LASSO) regression was used for variable selection when necessary. Predictive performance was assessed using receiver operating characteristic (ROC) curves and area under the curve (AUC). The optimal SLCVLTR cutoff was determined using the Youden index, and sensitivity, specificity, positive predictive value (PPV), and negative predictive value (NPV) were calculated. Differences in AUC between models were compared using the DeLong test. Model calibration was assessed using calibration curves and the Hosmer–Lemeshow goodness-of-fit test, and clinical net benefit was evaluated using decision curve analysis (DCA).

Because choroidal thickness may differ by sex, ROC analyses stratified by sex were performed, and an SLCVLTR × sex interaction term was added to the multivariable logistic regression model. To evaluate the influence of baseline disease severity, an extended severity model was constructed; analyses stratified by CST tertiles, an SLCVLTR × CST tertile interaction test, net reclassification improvement (NRI), and integrated discrimination improvement (IDI) analyses were performed.

Two graders with more than 5 years of OCT grading experience who were masked to clinical outcomes independently completed all manual measurements. Sixty eyes were randomly selected for repeatability assessment; the same grader repeated the measurements after 4 weeks. Interobserver and intraobserver agreement for continuous imaging parameters was assessed using the two-way random-effects absolute-agreement intraclass correlation coefficient (ICC), and agreement for categorical variables was assessed using the Cohen kappa (*κ*) coefficient (23). Model construction included candidate variables confirmed by clinical relevance and univariate screening, and the incremental predictive value of SLCVLTR beyond existing clinical and retinal OCT parameters was evaluated (24). Conservative classification was applied to eyes receiving rescue treatment before 6 months, and an exclusion sensitivity analysis was performed to reduce the influence of post-treatment OCT improvement on the determination of spontaneous resolution (25).

## 3 Results

### 3.1 General characteristics and rescue treatment handling

A total of 302 patients with T2DM-related DME (302 eyes) were included. At 6 months, 83 eyes (27.5%) met the criteria for spontaneous resolution, and 219 eyes (72.5%) were assigned to the non-resolution group. In the non-resolution group, 36 eyes received rescue treatment before 6 months because of DME progression, including anti-VEGF injections in 31 eyes, intravitreal corticosteroid therapy in 3 eyes, and macular laser therapy in 2 eyes; these eyes were assigned to the non-resolution group according to the prespecified rule.

### 3.2 Comparison of baseline clinical characteristics

There were no statistically significant differences between the spontaneous resolution and non-resolution groups in age, sex, BMI, history of hypertension, history of dyslipidemia, renal function indices, or lipid profile (all *p* > 0.05). Compared with the non-resolution group, the spontaneous resolution group had a shorter diabetes duration, lower HbA1c, lower fasting plasma glucose, a lower proportion of insulin use, and lower UACR; all these differences were statistically significant (all *p* < 0.05) (Table 1).

**Table 1:** Baseline clinical characteristics of the two groups.

Parameter	Spontaneous resolution group (*n* = 83)	Non-resolution group (*n* = 219)	Statistic	*p* value
Age (years, mean ± SD)	56.9 ± 9.8	58.1 ± 9.5	*t* = −0.971	0.332
Sex (male/female, *n*)	44/39	124/95	*χ^2^* = 0.318	0.573
BMI (kg/m^2^, mean ± SD)	24.8 ± 3.2	25.2 ± 3.5	*t* = −0.907	0.365
Diabetes duration (years, median [Q1, Q3])	8.0 (5.0, 12.0)	10.0 (6.0, 15.0)	*Z* = −2.305	0.021
HbA1c (%, mean ± SD)	7.4 ± 1.2	8.0 ± 1.4	*t* = −3.452	<0.001
Fasting plasma glucose (mmol/L, mean ± SD)	7.8 ± 2.1	8.5 ± 2.4	*t* = −2.339	0.020
Insulin use (*n* [%])	31 (37.3)	110 (50.2)	*χ^2^* = 4.011	0.045
History of hypertension (*n* [%])	42 (50.6)	121 (55.3)	*χ^2^* = 0.524	0.469
History of dyslipidemia (*n* [%])	28 (33.7)	82 (37.4)	*χ^2^* = 0.357	0.550
Serum creatinine (μmol/L, mean ± SD)	72.4 ± 18.6	75.1 ± 20.3	*t* = −1.055	0.292
Blood urea nitrogen (mmol/L, mean ± SD)	5.8 ± 1.7	6.1 ± 1.9	*t* = −1.260	0.209
Total cholesterol (mmol/L, mean ± SD)	4.7 ± 1.1	4.9 ± 1.2	*t* = −1.322	0.187
Triglycerides (mmol/L, median [Q1, Q3])	1.5 (1.0, 2.2)	1.6 (1.1, 2.4)	*Z* = −1.127	0.260
LDL-C (mmol/L, mean ± SD)	2.8 ± 0.8	2.9 ± 0.9	*t* = −0.888	0.375
HDL-C (mmol/L, mean ± SD)	1.2 ± 0.4	1.1 ± 0.4	*t* = 1.940	0.053
UACR (mg/g, median [Q1, Q3])	28.5 (12.0, 68.0)	45.2 (18.0, 112.0)	*Z* = −2.648	0.008

Continuous variables are presented as mean ± standard deviation (SD) or median (first quartile, third quartile) [median (Q1, Q3)] according to distribution; categorical variables are presented as counts (percentages) [*n* (%)]. *t* is the independent-samples *t*-test statistic, *Z* is the Mann–Whitney *U*-test statistic, and *χ*^2^ is the chi-square test statistic. BMI, body mass index; HbA1c, hemoglobin A1c; LDL-C, low-density lipoprotein cholesterol; HDL-C, high-density lipoprotein cholesterol; UACR, urinary albumin-to-creatinine ratio. *n*, number of patients or eyes; Q1, first quartile; Q3, third quartile. *p* values are two-sided.

### 3.3 Comparison of baseline ophthalmic examination and OCT parameters

There were no statistically significant differences between the two groups in intraocular pressure, lens status, or history of PRP (all *p* > 0.05). The spontaneous resolution group had better baseline BCVA, lower CST, a higher proportion of nonproliferative diabetic retinopathy (NPDR), a higher proportion of diffuse retinal thickening and a lower proportion of cystoid macular edema, higher proportions of intact EZ continuity and intact ELM, fewer HRF, a lower frequency of DRIL, and lower SFCT, SLCVLT, and SLCVLTR than the non-resolution group; all these differences were statistically significant (all *p* < 0.05) (Table 2).

**Table 2:** Baseline ophthalmic examination and optical coherence tomography parameters in the two groups.

Parameter	Spontaneous resolution group (*n* = 83)	Non-resolution group (*n* = 219)	Statistic	*P* value
BCVA (LogMAR, mean ± SD)	0.38 ± 0.18	0.46 ± 0.22	*t* = −2.958	0.003
Intraocular pressure (mmHg, mean ± SD)	15.2 ± 2.8	15.5 ± 3.0	*t* = −0.790	0.430
Lens status (clear crystalline lens/pseudophakia, *n*)	62/21	167/52	*χ^2^* = 0.080	0.778
DR stage (NPDR/PDR, *n*)	58/25	122/97	*χ^2^* = 5.020	0.025
History of PRP (*n* [%])	18 (21.7)	60 (27.4)	*χ^2^* = 1.025	0.311
CST (μm, mean ± SD)	368.5 ± 52.7	421.3 ± 78.4	*t* = −5.667	<0.001
Macular edema subtype (DRT/CME/SRD, *n*)	48/22/13	89/93/37	*χ*^2^ = 8.003	0.018
Intact EZ continuity (*n* [%])	59 (71.1)	115 (52.5)	*χ^2^* = 8.502	0.004
Intact ELM (*n* [%])	61 (73.5)	126 (57.5)	*χ^2^* = 6.502	0.011
Number of HRF (*n*, median [Q1, Q3])	4 (2, 7)	8 (4, 13)	*Z* = −4.718	<0.001
DRIL (*n* [%])	19 (22.9)	83 (37.9)	*χ^2^* = 6.061	0.014
SFCT (μm, mean ± SD)	248.6 ± 58.3	265.7 ± 62.1	*t* = −2.172	0.031
SLCVLT (μm, mean ± SD)	138.2 ± 36.4	155.8 ± 42.7	*t* = −3.324	0.001
SLCVLTR (%, mean ± SD)	55.1 ± 7.8	58.5 ± 6.9	*t* = −3.685	<0.001

Continuous variables are presented as mean ± SD or median (Q1, Q3); categorical variables are presented as *n* (%) or counts. *t* is the independent-samples *t*-test statistic, *Z* is the Mann–Whitney *U*-test statistic, and *χ*^2^ is the chi-square test statistic. OCT, optical coherence tomography; BCVA, best-corrected visual acuity; LogMAR, logarithm of the minimum angle of resolution; DR, diabetic retinopathy; NPDR, nonproliferative diabetic retinopathy; PDR, proliferative diabetic retinopathy; PRP, panretinal photocoagulation; CST, central subfield thickness; DRT, diffuse retinal thickening; CME, cystoid macular edema; SRD, serous retinal detachment; EZ, ellipsoid zone; ELM, external limiting membrane; HRF, hyperreflective foci; DRIL, disorganization of the retinal inner layers; SFCT, subfoveal choroidal thickness; SLCVLT, subfoveal large choroidal vessel layer thickness; SLCVLTR, subfoveal large choroidal vessel layer thickness ratio. *n*, number of eyes or counts; Q1, first quartile; Q3, third quartile. *p* values are two-sided.

### 3.4 Univariate and multivariable logistic regression analyses

Univariate logistic regression showed that diabetes duration, HbA1c, fasting plasma glucose, insulin use, UACR, BCVA, CST, DR stage, cystoid macular edema subtype, EZ continuity, ELM integrity, number of HRF, DRIL, SFCT, SLCVLT, and SLCVLTR were associated with spontaneous resolution of DME (all *p* < 0.10) (Supplementary Table 1).

Multivariable logistic regression showed that HbA1c, CST, cystoid macular edema subtype, number of HRF, and SLCVLTR were independent negative predictors of spontaneous resolution of DME, whereas intact EZ continuity was an independent positive predictor. The serous retinal detachment subtype and DRIL did not reach statistical significance (Table 3).

**Table 3:** Multivariable logistic regression analysis of factors associated with spontaneous resolution of diabetic macular edema.

Variable	*β*	SE	Wald *χ^2^*	OR (95% CI)	*P* value
HbA1c	−0.272	0.124	4.812	0.762 (0.597–0.971)	0.028
CST	−0.011	0.003	13.444	0.989 (0.983–0.995)	<0.001
Macular edema subtype (cystoid vs. diffuse)	−0.826	0.324	6.499	0.438 (0.232–0.826)	0.011
Macular edema subtype (serous retinal detachment vs. diffuse)	−0.287	0.378	0.576	0.751 (0.358–1.574)	0.448
EZ continuity (intact vs. disrupted)	0.618	0.301	4.215	1.855 (1.028–3.347)	0.040
Number of HRF	−0.082	0.028	8.577	0.921 (0.872–0.973)	0.003
DRIL (present vs. absent)	−0.438	0.312	1.971	0.645 (0.350–1.189)	0.160
SLCVLTR	−0.070	0.021	11.111	0.932 (0.895–0.972)	0.001

*β* is the regression coefficient. DME, diabetic macular edema; SE, standard error; Wald *χ*^2^, Wald chi-square statistic; OR, odds ratio; CI, confidence interval; HbA1c, hemoglobin A1c; CST, central subfield thickness; EZ, ellipsoid zone; HRF, hyperreflective foci; DRIL, disorganization of the retinal inner layers; SLCVLTR, subfoveal large choroidal vessel layer thickness ratio. Logistic regression used spontaneous resolution of DME as the dependent variable. CST, HbA1c, number of HRF, and SLCVLTR were included as continuous variables; macular edema subtype used diffuse retinal thickening as the reference category; EZ continuity used disruption as the reference category; DRIL used absence as the reference category. vs. indicates comparison with the reference category. *p* values are two-sided.

### 3.5 Clinical-OCT combined model and incremental predictive performance of SLCVLTR

**Figure 2:**
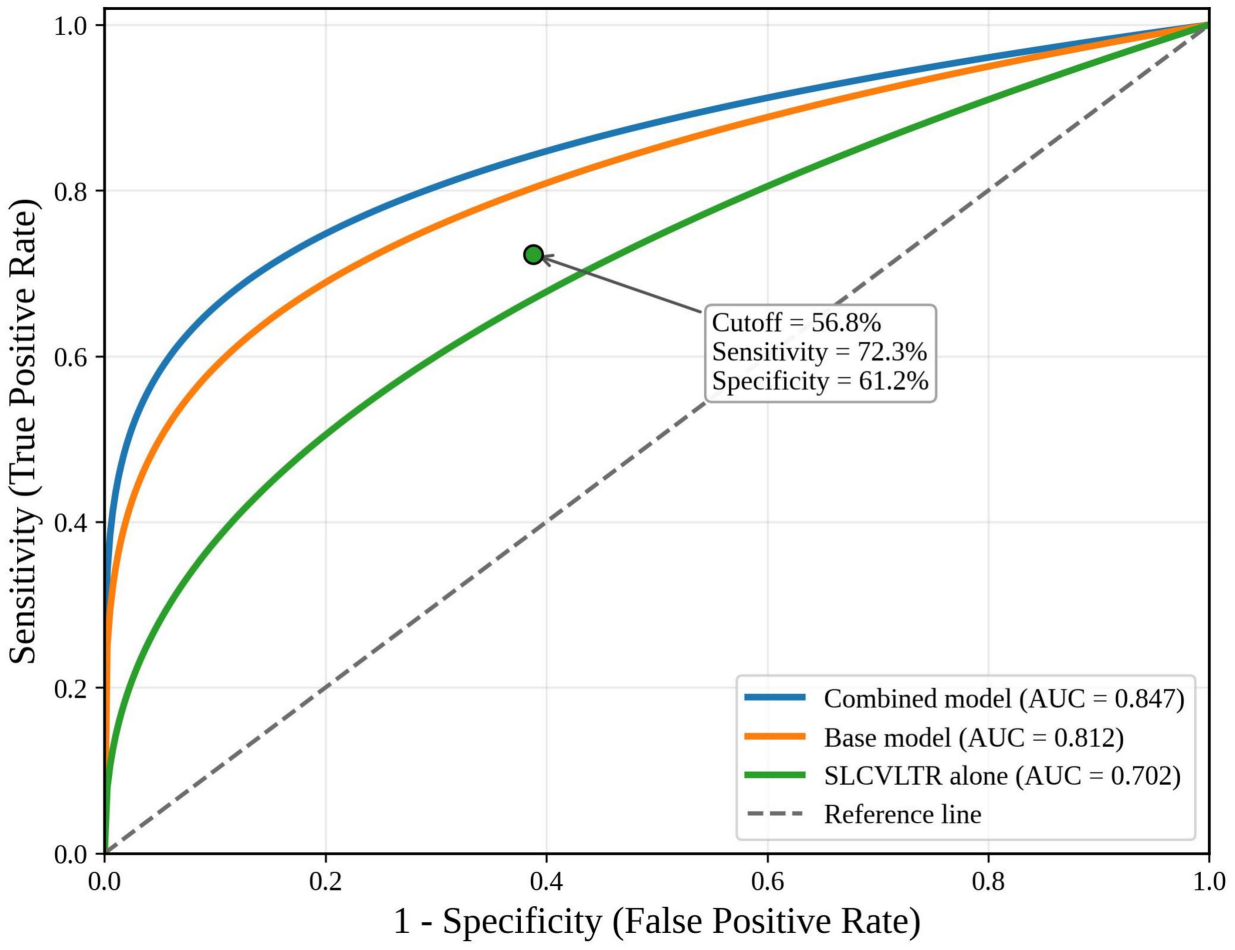
Receiver operating characteristic (ROC) curves of prediction models for spontaneous resolution of diabetic macular edema (DME). The curves show the discriminatory performance of the subfoveal large choroidal vessel layer thickness ratio (SLCVLTR)-alone model, base model, and combined model for 6-month spontaneous resolution of DME. The combined model consisted of hemoglobin A1c (HbA1c), central subfield thickness (CST), macular edema subtype, ellipsoid zone (EZ) continuity, number of hyperreflective foci (HRF), and SLCVLTR; the base model did not include SLCVLTR. The optimal SLCVLTR cutoff of 56.8%, sensitivity of 72.3%, and specificity of 61.2% are labeled. DME, diabetic macular edema; ROC, receiver operating characteristic; AUC, area under the curve; SLCVLTR, subfoveal large choroidal vessel layer thickness ratio; HbA1c, hemoglobin A1c; CST, central subfield thickness; EZ, ellipsoid zone; HRF, hyperreflective foci. Sensitivity (True Positive Rate) = sensitivity (true-positive rate); 1-Specificity (False Positive Rate) = 1-specificity (false-positive rate); Combined model = combined model; Base model = base model; SLCVLTR alone = SLCVLTR-alone model; Reference line = reference line; Cutoff = cutoff; Sensitivity = sensitivity; Specificity = specificity.

**Figure 3:**
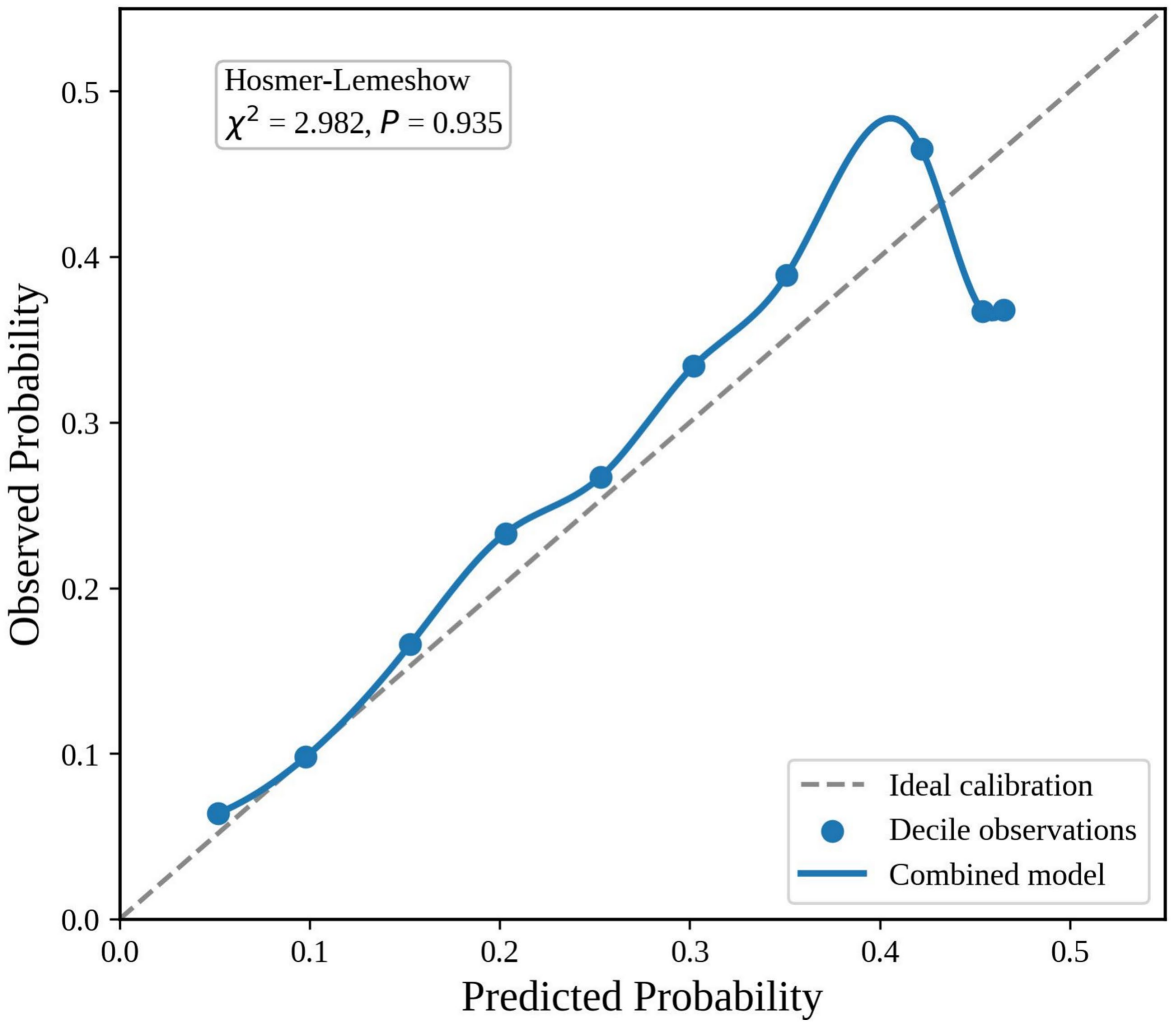
Calibration curve of the combined prediction model. The *x*-axis represents the predicted probability of spontaneous resolution, and the *y*-axis represents the observed probability of spontaneous resolution. The calibration curve is close to the ideal calibration line, and the Hosmer–Lemeshow test (*χ*^2^ = 2.982, *p* = 0.935) suggests good agreement between predicted and observed probabilities. Predicted Probability = predicted probability; Observed Probability = observed probability; Ideal calibration = ideal calibration; Decile observations = decile observations; Combined model = combined model.

**Figure 4:**
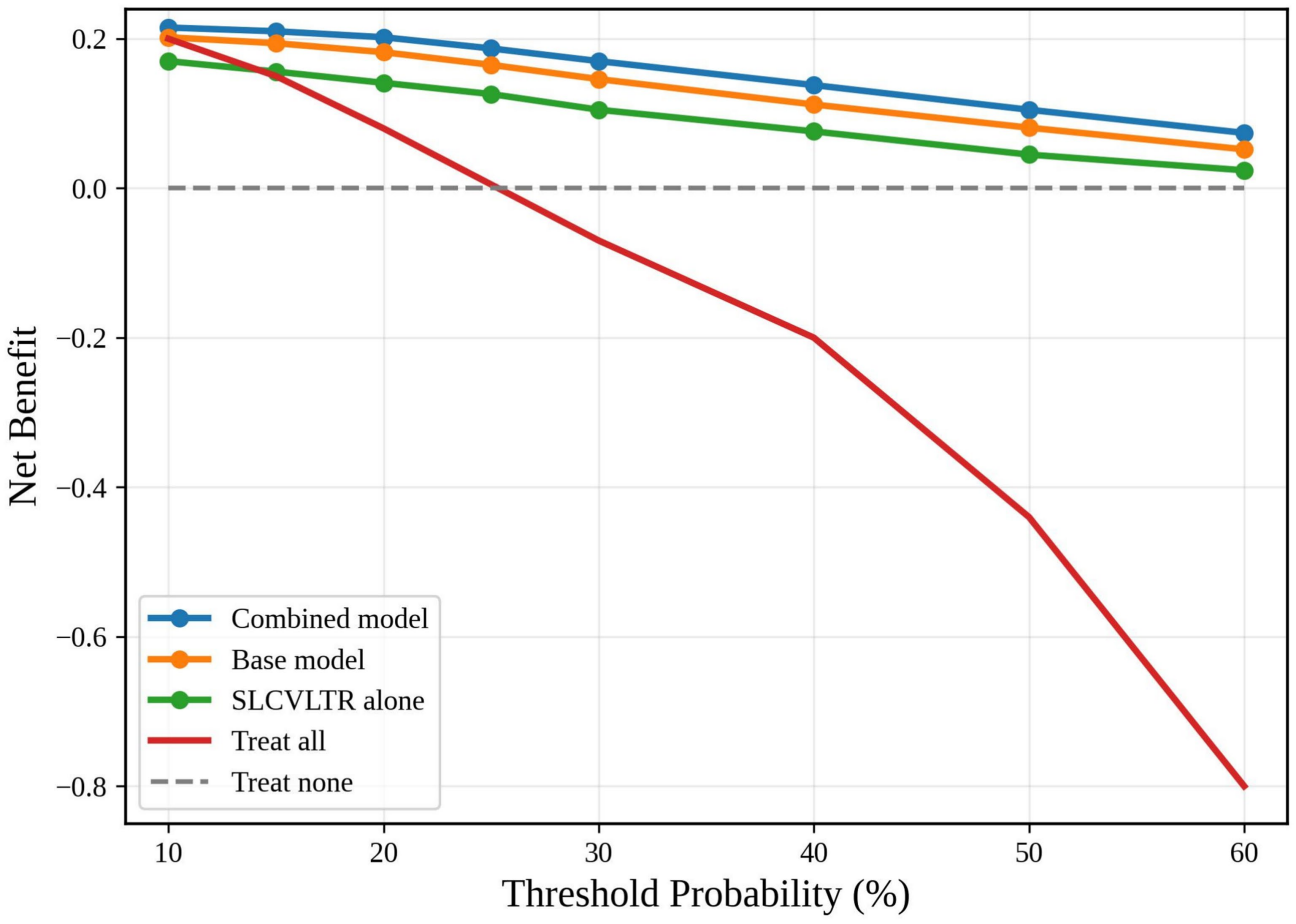
Decision curve analysis showed that the combined model provided the highest net benefit across the evaluated threshold probability range of 10–60%, including the clinically relevant range of 15–50%, compared with the SLCVLTR-alone model, base model, treat-all strategy, and treat-none strategy. SLCVLTR, subfoveal large choroidal vessel layer thickness ratio. Net benefit = net benefit; Threshold probability (%) = threshold probability (%); Combined model = combined model; Base model = base model; SLCVLTR alone = SLCVLTR-alone model; Treat all = treat all; Treat none = treat none.

The SLCVLTR-alone model for predicting spontaneous resolution of DME had an AUC of 0.702 (95% CI, 0.638–0.766). The optimal cutoff was 56.8%, with a sensitivity of 72.3% and specificity of 61.2%. The combined model consisting of HbA1c, CST, macular edema subtype, EZ continuity, number of HRF, and SLCVLTR had an AUC of 0.847 (95% CI, 0.800–0.894), which was higher than that of the SLCVLTR-alone model (*p* < 0.001) and the base model without SLCVLTR (*p* = 0.027). The combined model showed good calibration (Hosmer–Lemeshow *χ*^2^ = 2.982, *p* = 0.935) and provided the highest net benefit across the evaluated threshold probability range of 10–60%, including the clinically relevant range of 15–50% (Table 4, Figures 2–4, Supplementary Tables 2, 3).

**Table 4:** Summary of receiver operating characteristic performance, calibration, and clinical net benefit of prediction models.

Model/metric	AUC (95% CI)	Optimal cutoff	Sensitivity (%)	Specificity (%)	PPV (%)	NPV (%)	Additional test
SLCVLTR-alone model	0.702 (0.638–0.766)	56.8%	72.3	61.2	41.4	85.4	Reference
Base model	0.812 (0.761–0.863)	–	74.7	73.5	51.7	88.5	Does not include SLCVLTR
Combined model	0.847 (0.800–0.894)	–	78.3	76.7	56.0	90.3	Compared with SLCVLTR: *P* < 0.001; compared with base model: *P* = 0.027
Calibration	–	–	–	–	–	–	Hosmer–Lemeshow *χ^2^* = 2.982, df = 8, *P* = 0.935
Decision curve	–	–	–	–	–	–	The combined model provided the highest net benefit across the evaluated threshold probability range of 10–60%, including the clinically relevant range of 15–50%.

ROC, receiver operating characteristic; AUC, area under the curve; CI, confidence interval; PPV, positive predictive value; NPV, negative predictive value; SLCVLTR, subfoveal large choroidal vessel layer thickness ratio; HbA1c, hemoglobin A1c; CST, central subfield thickness; EZ, ellipsoid zone; HRF, hyperreflective foci; DCA, decision curve analysis. The base model included HbA1c, CST, macular edema subtype, EZ continuity, and number of HRF; the combined model added SLCVLTR to the base model. AUC comparisons between models were performed using the DeLong test; model calibration was assessed using the Hosmer–Lemeshow goodness-of-fit test; clinical net benefit was evaluated using DCA. df, degrees of freedom; Hosmer–Lemeshow *χ*^2^ is the goodness-of-fit test statistic. *P* values are two-sided.

### 3.6 Sex-stratified, treatment-handling, and disease-severity sensitivity analyses

Optimal SLCVLTR cutoffs were similar in men and women, and the between-sex difference in AUC was not statistically significant; the SLCVLTR × sex interaction term was also not statistically significant. A total of 22 eyes underwent peripheral PRP within 6 months because of PDR or high-risk PDR, with no statistically significant difference between the spontaneous resolution and non-resolution groups. After excluding cases that underwent PRP during follow-up or excluding eyes that received rescue treatment for DME, SLCVLTR remained independently associated with spontaneous resolution. In the extended severity model including HbA1c, BCVA, CST, DR stage, macular edema subtype, EZ, ELM, HRF, and DRIL, SLCVLTR retained an independent effect. Adding SLCVLTR yielded a statistically significant incremental increase in discriminatory ability compared with the base model. Across CST tertiles, the direction of the SLCVLTR effect was consistent, and the SLCVLTR × CST tertile interaction term did not reach statistical significance (Table 5).

**Table 5:** Sex-stratified analysis, treatment handling, severity adjustment, and sensitivity analyses.

Analysis	Sample/subgroup	Estimate	*P* value	Remarks
Male SLCVLTR ROC	*n* = 168; spontaneous resolution 44, non-resolution 124	AUC 0.696 (0.608–0.784); cutoff 57.1%	–	Sensitivity 70.5%, specificity 60.5%, PPV 38.8%, NPV 85.2%
Female SLCVLTR ROC	*n* = 134; spontaneous resolution 39, non-resolution 95	AUC 0.710 (0.615–0.805); cutoff 56.2%	–	Sensitivity 74.4%, specificity 62.1%, PPV 44.6%, NPV 85.5%
Male vs. female AUC comparison	*n* = 302	DeLong *p* = 0.817	0.817	No statistically significant between-sex difference in stratified AUC
SLCVLTR × sex	*n* = 302	OR 1.006 (0.974–1.039)	0.721	Interaction term not statistically significant
New PRP during follow-up	*n* = 302; spontaneous resolution 4/83, non-resolution 18/219	22/302 (7.3%); *χ^2^* = 1.030	0.310	No statistically significant between-group difference
After excluding PRP during follow-up	*n* = 280	SLCVLTR OR 0.929 (0.890–0.971)	0.001	Combined-model AUC 0.844 (0.793–0.895)
Rescue treatment composition	*n* = 36; all assigned to non-resolution group	Anti-VEGF 31; corticosteroid 3; macular laser 2	–	Most recent pretreatment OCT was used as evidence of disease progression
After excluding rescue treatment	*n* = 266	SLCVLTR OR 0.936 (0.896–0.978)	0.003	Combined-model AUC 0.833 (0.778–0.889)
Extended severity model	*n* = 302	SLCVLTR OR 0.936 (0.897–0.977)	0.003	Adjusted for HbA1c, BCVA, CST, DR stage, DME subtype, EZ, ELM, HRF, and DRIL
Base model vs. combined model	*n* = 302	ΔAUC = 0.035 (0.812 to 0.847)	0.027	DeLong test
Continuous NRI	*n* = 302	0.214	0.018	Reclassification improvement after adding SLCVLTR
IDI	*n* = 302	0.041	0.006	Improvement in mean discrimination slope after adding SLCVLTR
Lowest CST tertile	*n* = 101; ≤370 μm	SLCVLTR OR 0.931 (0.872–0.994)	0.032	Direction of stratified effect was consistent
Middle CST tertile	*n* = 101; 371–430 μm	SLCVLTR OR 0.936 (0.886–0.988)	0.017	Direction of stratified effect was consistent
Highest CST tertile	*n* = 100; >430 μm	SLCVLTR OR 0.947 (0.896–1.002)	0.059	Direction of effect was consistent, but statistical significance was borderline
SLCVLTR × CST tertile	*n* = 302	Interaction *p* = 0.642	0.642	No significant difference in effect across CST strata

AUC, area under the curve; ROC, receiver operating characteristic; OR, odds ratio; CI, confidence interval; PPV, positive predictive value; NPV, negative predictive value; PRP, panretinal photocoagulation; VEGF, vascular endothelial growth factor; OCT, optical coherence tomography; DME, diabetic macular edema; HbA1c, hemoglobin A1c; BCVA, best-corrected visual acuity; CST, central subfield thickness; DR, diabetic retinopathy; EZ, ellipsoid zone; ELM, external limiting membrane; HRF, hyperreflective foci; DRIL, disorganization of the retinal inner layers; SLCVLTR, subfoveal large choroidal vessel layer thickness ratio; NRI, net reclassification improvement; IDI, integrated discrimination improvement; ΔAUC indicates the difference in area under the curve. AUC comparisons were performed using the DeLong test, effect estimates were obtained using logistic regression, and interaction tests were used to evaluate sex- or CST-stratified effects. *n*, sample size; *χ*^2^, chi-square statistic; vs. indicates comparison between the two models. *P* values are two-sided.

### 3.7 Reliability of imaging measurements

**Figure 5:**
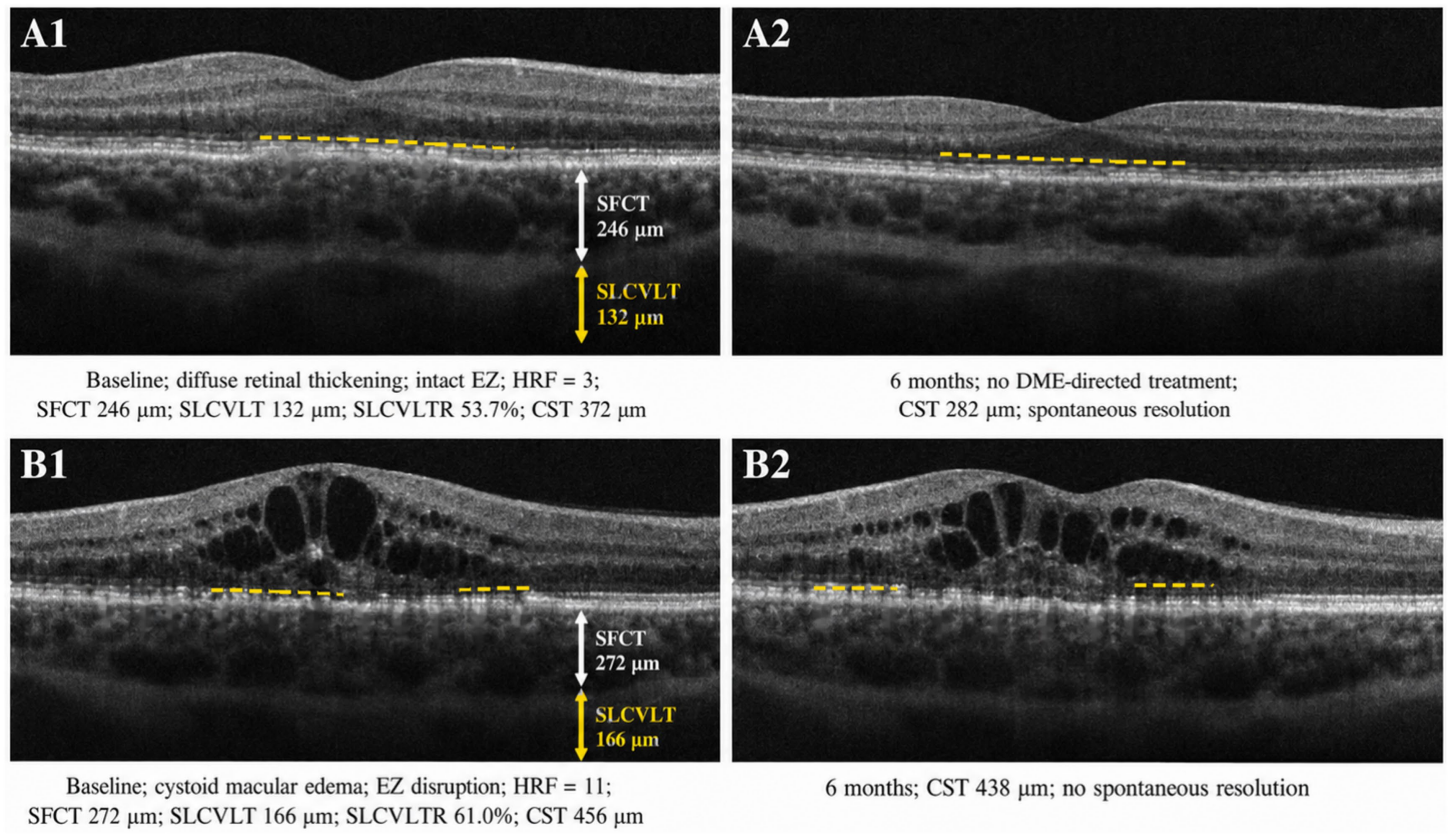
Representative enhanced-depth imaging optical coherence tomography (EDI-OCT) images of the spontaneous resolution and non-resolution groups. (A1, A2) Representative spontaneous resolution case, a 59-year-old man with diffuse retinal thickening at baseline, continuous central macular ellipsoid zone (EZ) assessment region, hyperreflective foci (HRF) count of 3, subfoveal choroidal thickness (SFCT) of 246 μm, subfoveal large choroidal vessel layer thickness (SLCVLT) of 132 μm, subfoveal large choroidal vessel layer thickness ratio (SLCVLTR) of 53.7%, and baseline central subfield thickness (CST) of 372 μm; he received no diabetic macular edema (DME)-directed treatment within 6 months, and CST decreased to 282 μm, meeting the criteria for spontaneous resolution. (B1, B2) Representative non-resolution case, a 61-year-old woman with cystoid macular edema at baseline, disrupted central macular EZ assessment region, HRF count of 11, SFCT of 272 μm, SLCVLT of 166 μm, SLCVLTR of 61.0%, and baseline CST of 456 μm; CST at 6 months was 438 μm, not meeting the criteria for spontaneous resolution. White double-headed arrows indicate SFCT, yellow double-headed arrows indicate SLCVLT, and yellow dashed short segments indicate the central macular EZ assessment region. HRF counts were obtained from punctate hyperreflective foci within the central 3-mm neurosensory retina on the horizontal B-scan passing through the fovea, with a maximum diameter ≤30 μm and no obvious posterior shadowing; SFCT and SLCVLT were measured according to structural boundaries in the choroidal layer. CST, central subfield thickness; DME, diabetic macular edema; EDI-OCT, enhanced-depth imaging optical coherence tomography; EZ, ellipsoid zone; HRF, hyperreflective foci; SFCT, subfoveal choroidal thickness; SLCVLT, subfoveal large choroidal vessel layer thickness; SLCVLTR, subfoveal large choroidal vessel layer thickness ratio. Baseline = baseline; 6 months = 6 months; no DME-directed treatment = no DME-directed treatment; spontaneous resolution = spontaneous resolution; no spontaneous resolution = no spontaneous resolution; diffuse retinal thickening = diffuse retinal thickening; intact EZ = intact EZ; cystoid macular edema = cystoid macular edema; EZ disruption = EZ disruption.

Among 60 randomly selected eyes, SFCT, SLCVLT, SLCVLTR, and manually assessed OCT biomarkers all showed good interobserver and intraobserver agreement (Supplementary Table 4). Representative EDI-OCT images of the spontaneous resolution and non-resolution groups showed typical imaging differences in macular edema morphology, EZ continuity, HRF burden, SFCT, SLCVLT, and SLCVLTR between the two groups (Figure 5).

## 4 Discussion

This study showed that spontaneous resolution of DME was jointly associated with systemic metabolic control, baseline edema severity, retinal microstructural integrity, and choroidal laminar structure. Higher HbA1c, higher CST, cystoid macular edema subtype, greater number of HRF, and higher SLCVLTR were associated with a lower probability of spontaneous resolution, whereas intact EZ continuity was associated with a higher probability of spontaneous resolution. These findings are consistent with evidence on OCT grading and inflammation-related imaging biomarkers (26). Existing evidence also indicates that baseline cystoid spaces, HRF, outer retinal integrity, and other OCT features are associated with subsequent anatomical recurrence or treatment response (27).

Systemic metabolic status is an important contextual determinant of the DME disease course. Higher HbA1c may reflect chronic hyperglycemia-related endothelial injury, inflammatory activation, and blood-retinal barrier dysfunction; existing evidence shows that metabolic risk factors are closely associated with the occurrence and progression of DME (28). The association between peripheral inflammatory markers and anti-VEGF treatment response further suggests that systemic inflammation and the background of diabetic microvascular disease may influence imaging and anatomical outcomes in DME (29).

CST and macular edema subtype remain core indicators of anatomical severity. Higher CST indicates a greater edema burden, and cystoid macular edema suggests more pronounced fluid accumulation and impaired glial support structures. Wide-field OCT evidence shows that extramacular structural abnormalities and central macular changes may jointly characterize disease burden in patients with DME (30). SLCVLTR retained an independent effect in the extended severity model, suggesting that it may provide complementary characterization beyond CST and related to the outer retinal/choroidal microenvironment. Evidence from three-dimensional assessment of choroidal vessels also supports the presence of choroidal vascular structural changes in diabetic retinopathy that are independent of conventional retinal thickness parameters (31).

The clinical interpretation of SLCVLTR should focus on its role as a candidate prognostic imaging biomarker and a source of incremental information. Higher SLCVLTR may represent relative expansion of Haller’s layer or an increased proportion of the large choroidal vessel layer, reflecting differences in choroidal laminar remodeling, the local hydrostatic environment, and fluid transport capacity around the RPE-Bruch’s membrane complex. Current evidence indicates that treatment strategies remain based on interventions targeting vascular permeability and inflammatory burden, including anti-VEGF therapy, corticosteroids, and laser therapy (32). Advances in automated OCT quantification and imaging algorithms make risk stratification based on multidimensional imaging features a potential future application (33).

The combined model outperformed the SLCVLTR-alone model and the base model without SLCVLTR, indicating that a multidimensional parameter combination better reflects the complexity of the natural course of DME. New dual-pathway agents and longer-interval treatment regimens are reducing treatment burden, but individualized follow-up and the timing of treatment initiation still depend on accurate baseline risk assessment (34). Evidence from long-term treatment management also emphasizes the need to balance reduction in injection burden with maintenance of anatomical stability (35).

HRF are associated with inflammation and lipid exudation burden. In this study, a higher HRF count was associated with a lower likelihood of spontaneous resolution. Changes in local inflammation-related OCT features before and after anti-VEGF therapy suggest that HRF may serve as an imaging reflection of DME activity and microenvironmental changes (36). Evidence regarding systemic predictive factors further supports incorporating blood glucose, renal function, and other metabolic indicators into the DME risk assessment framework (37).

DR stage and DRIL were associated with outcomes in univariate analysis but did not retain independent statistical significance in the final model. The overall severity of retinopathy represented by DR stage may be related to neurovascular unit dysfunction and the background of microvascular injury (38). Previous DME studies have shown that the horizontal extent and dynamic changes of DRIL within the central 1-mm foveal region are closely related to visual function, and involvement of at least 50% of the central 1-mm region (approximately 500 μm) by DRIL can serve as a clinically meaningful criterion for defining foveal DRIL (19–21, 39). In this study, the independent effect of DRIL may have been partially explained by CST, EZ continuity, and HRF burden. The imaging measurement reliability results showed high repeatability of SLCVLT, SLCVLTR, and manually assessed OCT biomarkers, consistent with the feasibility supported by evidence on choroidal segmentation measurements (40).

Clinically, the combined model can be used for risk stratification under initial observation conditions. For patients with relatively stable visual acuity, the ability to undergo close follow-up, and a high predicted probability of spontaneous resolution according to the combined model, short-term observation with optimization of systemic metabolic control may be clinically reasonable. For patients with higher CST, cystoid edema, EZ disruption, high HRF burden, or elevated SLCVLTR, higher-risk stratification may support closer follow-up and earlier initiation of DME-directed treatment. Evidence regarding the initial observation strategy indicates that close follow-up and prespecified rescue treatment trigger criteria are important prerequisites for safe implementation of observation (41).

This study has several limitations. The retrospective single-center design may introduce selection bias. The study population focused on T2DM-related DME; therefore, model applicability is mainly limited to this population and requires validation in type 1 diabetes and other diabetes-related DME populations. Future studies should include external validation, automated segmentation assessment, and integration of OCTA perfusion parameters.

## 5 Conclusion

Spontaneous resolution of DME was jointly associated with systemic metabolic control, baseline edema severity, retinal microstructural integrity, and choroidal laminar structure. Lower HbA1c, lower CST, noncystoid macular edema morphology, intact EZ continuity, fewer HRF, and lower SLCVLTR indicated a higher probability of spontaneous resolution. As a candidate choroidal imaging biomarker, SLCVLTR provided independent and incremental predictive value beyond existing clinical and retinal OCT parameters. The combined model may provide a basis for risk stratification in patients with DME who meet criteria for initial observation and support individualized follow-up and treatment decision-making.

## Data Availability

The original contributions presented in the study are included in the article/Supplementary material, further inquiries can be directed to the corresponding author.
