# Supplementary material for: Clinical and optical coherence tomography predictors of spontaneous resolution of diabetic macular edema: incremental value of the subfoveal large choroidal vessel layer thickness ratio
Source: Front Med (Lausanne). 2026 Jul 30;13:1901761. doi: 10.3389/fmed.2026.1901761 (PMC13470262; doi:10.3389/fmed.2026.1901761)
Supplement: Supplementary file 1 [file Table_1.DOCX]

# **Supplementary Material**

**Supplementary Table 1. Univariate logistic regression analysis**

| **Variable** | ***β*** | **SE** | **Wald *χ²*** | **OR (95% CI)** | ***P* value** |
| --- | --- | --- | --- | --- | --- |
| Diabetes duration | -0.058 | 0.025 | 5.382 | 0.944 (0.899-0.991) | 0.020 |
| HbA1c | -0.328 | 0.111 | 8.732 | 0.720 (0.579-0.895) | 0.003 |
| Fasting plasma glucose | -0.136 | 0.056 | 5.898 | 0.873 (0.782-0.974) | 0.015 |
| Insulin use (yes vs. no) | -0.527 | 0.255 | 4.271 | 0.590 (0.358-0.973) | 0.039 |
| UACR | -0.004 | 0.0016 | 6.250 | 0.996 (0.993-0.999) | 0.012 |
| BCVA (LogMAR) | -2.145 | 0.812 | 6.978 | 0.117 (0.024-0.575) | 0.008 |
| CST | -0.013 | 0.003 | 18.778 | 0.987 (0.981-0.993) | <0.001 |
| DR stage (PDR vs. NPDR) | -0.622 | 0.263 | 5.593 | 0.537 (0.321-0.899) | 0.018 |
| Macular edema subtype (cystoid vs. diffuse) | -0.765 | 0.280 | 7.465 | 0.465 (0.269-0.806) | 0.006 |
| Macular edema subtype (SRD vs. diffuse) | -0.345 | 0.348 | 0.983 | 0.708 (0.358-1.401) | 0.322 |
| EZ continuity (intact vs. disrupted) | 0.805 | 0.264 | 9.298 | 2.237 (1.333-3.753) | 0.002 |
| ELM integrity (intact vs. disrupted) | 0.708 | 0.268 | 6.979 | 2.030 (1.200-3.432) | 0.008 |
| Number of HRF | -0.108 | 0.024 | 20.250 | 0.898 (0.856-0.941) | <0.001 |
| DRIL (present vs. absent) | -0.722 | 0.284 | 6.463 | 0.486 (0.278-0.848) | 0.011 |
| SFCT | -0.005 | 0.002 | 6.250 | 0.995 (0.991-0.999) | 0.012 |
| SLCVLT | -0.011 | 0.004 | 7.562 | 0.989 (0.981-0.997) | 0.006 |
| SLCVLTR | -0.063 | 0.018 | 12.250 | 0.939 (0.906-0.973) | <0.001 |

Note: *β* is the univariate logistic regression coefficient. DME=diabetic macular edema; SE=standard error; Wald *χ²* = Wald chi-square statistic; OR=odds ratio; CI=confidence interval; HbA1c=hemoglobin A1c; UACR=urinary albumin-to-creatinine ratio; BCVA=best-corrected visual acuity; LogMAR=logarithm of the minimum angle of resolution; CST=central subfield thickness; DR=diabetic retinopathy; PDR=proliferative diabetic retinopathy; NPDR=nonproliferative diabetic retinopathy; SRD=serous retinal detachment; EZ=ellipsoid zone; ELM=external limiting membrane; HRF=hyperreflective foci; DRIL=disorganization of the retinal inner layers; SFCT=subfoveal choroidal thickness; SLCVLT=subfoveal large choroidal vessel layer thickness; SLCVLTR=subfoveal large choroidal vessel layer thickness ratio. Spontaneous resolution of DME was used as the dependent variable. vs. indicates comparison with the reference category. *P* values are two-sided.

**Supplementary Table 2. Hosmer-Lemeshow decile calibration details**

| **Decile** | **Observed n** | **Observed spontaneous resolution n** | **Predicted spontaneous resolution n** | **Observed non-resolution n** | **Predicted non-resolution n** |
| --- | --- | --- | --- | --- | --- |
| 1 | 30 | 2 | 1.6 | 28 | 28.4 |
| 2 | 31 | 3 | 3.1 | 28 | 27.9 |
| 3 | 30 | 5 | 4.6 | 25 | 25.4 |
| 4 | 30 | 7 | 6.1 | 23 | 23.9 |
| 5 | 30 | 8 | 7.6 | 22 | 22.4 |
| 6 | 30 | 10 | 9.1 | 20 | 20.9 |
| 7 | 31 | 12 | 10.8 | 19 | 20.2 |
| 8 | 30 | 14 | 12.6 | 16 | 17.4 |
| 9 | 30 | 11 | 13.6 | 19 | 16.4 |
| 10 | 30 | 11 | 13.9 | 19 | 16.1 |

Note: Model calibration was assessed using the Hosmer-Lemeshow test. Decile groups were ordered from low to high according to model-predicted probability; observed n indicates the sample size in each decile group; predicted counts indicate the model-predicted number of events or nonevents. Hosmer-Lemeshow *χ²* = 2.982, df = 8, *P* = 0.935. df = degrees of freedom; *P* values are two-sided.

**Supplementary Table 3. Net benefit details at different threshold probabilities**

| **Threshold probability (%)** | **Combined model** | **Base model** | **SLCVLTR-alone model** | **Treat all** | **Treat none** |
| --- | --- | --- | --- | --- | --- |
| 10 | 0.215 | 0.198 | 0.168 | 0.194 | 0 |
| 15 | 0.208 | 0.190 | 0.155 | 0.147 | 0 |
| 20 | 0.196 | 0.178 | 0.142 | 0.094 | 0 |
| 25 | 0.182 | 0.163 | 0.126 | 0.033 | 0 |
| 30 | 0.168 | 0.146 | 0.108 | -0.036 | 0 |
| 40 | 0.138 | 0.114 | 0.076 | -0.209 | 0 |
| 50 | 0.105 | 0.082 | 0.048 | -0.450 | 0 |
| 60 | 0.072 | 0.054 | 0.025 | -0.813 | 0 |

Note: Net benefit is presented as net benefit. Threshold probability was the decision threshold used by the model to determine a high probability of spontaneous resolution. SLCVLTR=subfoveal large choroidal vessel layer thickness ratio; HbA1c=hemoglobin A1c; CST=central subfield thickness; EZ=ellipsoid zone; HRF=hyperreflective foci. The combined model included HbA1c, CST, macular edema subtype, EZ continuity, number of HRF, and SLCVLTR; the base model did not include SLCVLTR. Net benefit for the treat-all strategy was calculated as: event rate - (1 - event rate) × threshold probability / (1 - threshold probability). Across all evaluated threshold probabilities from 10% to 60%, the combined model provided a higher net benefit than the base model, the SLCVLTR-alone model, the treat-all strategy, and the treat-none strategy.*P* values are two-sided.

**Supplementary Table 4. Agreement of choroidal measurements and manually assessed OCT biomarkers**

| **Indicator** | **Variable type** | **Interobserver agreement** | **Intraobserver agreement** | ***P* value** |
| --- | --- | --- | --- | --- |
| SFCT | Continuous variable | ICC = 0.943 (0.928-0.956) | ICC = 0.956 (0.944-0.966) | <0.001 |
| SLCVLT | Continuous variable | ICC = 0.927 (0.908-0.942) | ICC = 0.941 (0.925-0.954) | <0.001 |
| SLCVLTR | Continuous variable | ICC = 0.918 (0.897-0.936) | ICC = 0.932 (0.914-0.947) | <0.001 |
| CSI clearly discernible | Binary variable | *κ* = 0.889 | *κ* = 0.913 | <0.001 |
| DME subtype | Categorical variable | *κ* = 0.858 | *κ* = 0.884 | <0.001 |
| EZ continuity | Binary variable | *κ* = 0.823 | *κ* = 0.861 | <0.001 |
| ELM integrity | Binary variable | *κ* = 0.806 | *κ* = 0.842 | <0.001 |
| Number of HRF | Count variable | ICC = 0.892 (0.851-0.923) | ICC = 0.913 (0.879-0.939) | <0.001 |
| DRIL | Binary variable | *κ* = 0.814 | *κ* = 0.846 | <0.001 |

Note: OCT=optical coherence tomography; SFCT=subfoveal choroidal thickness; SLCVLT=subfoveal large choroidal vessel layer thickness; SLCVLTR=subfoveal large choroidal vessel layer thickness ratio; CSI=choroid-scleral interface; DME=diabetic macular edema; EZ=ellipsoid zone; ELM=external limiting membrane; HRF=hyperreflective foci; DRIL=disorganization of the retinal inner layers; ICC = intraclass correlation coefficient, used to evaluate agreement for continuous or count variables; *κ* = Cohen kappa coefficient, used to evaluate agreement for categorical variables. Interobserver agreement indicates agreement between the two graders; intraobserver agreement indicates agreement for repeated measurements by the same grader after 4 weeks. *P* values are two-sided.
